# Scleroderma

**Published:** 1890-03-08

**Authors:** 


### SKIN DEPARTMENT.

### SCLERODERMA.

The following rare case of scleroderma—there are only two hundred in all recorded from Germany—is at present an inmate of Zurich Hospital. M., a married woman, 45 years of age, mother of one child, stout, puffy in aspect, and of rather florid complexion, had occasion to do much washing during the winter of ten years ago. She contracted frequent colds at that time, the only cause to which she can attribute the gradually advancing thickening of the skin which has come upon her. This thickening is not œdematous or in nodules; but uniform, as if consequent upon an even development of connective tissue in the affected parts. It is principally confined to the head and upper extremities, with traces, however, also in the lower. The mouth can only be opened with difficulty, owing to thickening of the lips; and the tissues of eyelids and external ears are very abnormally developed. There are numerous warty growths on the hands, the nails are short and bent, and the balls of the fingers triple the normal size, forming globular swellings. The hands occasionally become cyanotic, as in cases of Raynaud's disease. Patient never suffered from rheumatism or hæmoglobinuria; has no cardiac murmur, and no signs and symptoms of myxœdema. Treatment by rest in bed, regimen, and salt-water baths has had no great measure of success, and the drugs tried have been without favourable result.

